# Recent advances in forensic anthropology: decomposition research

**DOI:** 10.1080/20961790.2018.1488571

**Published:** 2018-08-13

**Authors:** Daniel J. Wescott

**Affiliations:** Department of Anthropology, Texas State University, Forensic Anthropology Center at Texas State, San Marcos, TX, USA

**Keywords:** Taphonomy, postmortem interval, carrion ecology, decomposition

## Abstract

Decomposition research is still in its infancy, but significant advances have occurred within forensic anthropology and other disciplines in the past several decades. Decomposition research in forensic anthropology has primarily focused on estimating the postmortem interval (PMI), detecting clandestine remains, and interpreting the context of the scene. Additionally, while much of the work has focused on forensic-related questions, an interdisciplinary focus on the ecology of decomposition has also advanced our knowledge. The purpose of this article is to highlight some of the fundamental shifts that have occurred to advance decomposition research, such as the role of primary extrinsic factors, the application of decomposition research to the detection of clandestine remains and the estimation of the PMI in forensic anthropology casework. Future research in decomposition should focus on the collection of standardized data, the incorporation of ecological and evolutionary theory, more rigorous statistical analyses, examination of extended PMIs, greater emphasis on aquatic decomposition and interdisciplinary or transdisciplinary research, and the use of human cadavers to get forensically reliable data.

## Introduction

Laboratory-based identification of human skeletal remains has been the primary focus of forensic anthropology for much of the discipline’s history. This emphasis on identification is clearly reflected in journal publications beginning with the inception of forensic anthropology to the present that focus almost exclusively on the development and validation of methods for estimating biological characteristics (e.g. age-at-death, sex, ancestry, and stature) from the human skeleton. However, over the past several decades there has been an expansion of the role of forensic anthropologists in medicolegal death inquiries – with forensic anthropologists increasingly being invited to participate in scene recoveries to locate clandestine remains, provide contextual information at the scene, and to estimate the postmortem interval (PMI). As a result, there has also been a corresponding broadening of scientific questions under scrutiny by forensic anthropologists, including those related to human decomposition. As Dirkmaat et al. [1] noted, forensic taphonomy, including decomposition, provides “forensic anthropology with a new conceptual framework, which is broader, deeper, and more solidly entrenched in the natural sciences…” and “represents a true paradigm shift.”

**Figure 1.**
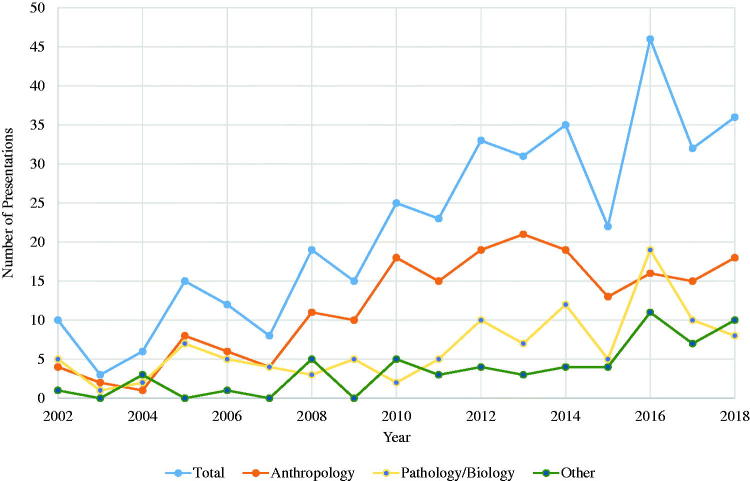
Trends in decomposition-related studies presented at the American Academy of Forensic Sciences from 2002 to 2018. Graph shows total number of papers presented and the number presented in the Anthropology, Pathology/Biology, and Other sections.

Not surprisingly, the desire for knowledge about the decomposition process and its applications to medicolegal death investigations has not only increased in forensic anthropology but in many other forensic science fields (e.g. entomology, pathology/biology, toxicology, and chemistry), and has resulted in an increase in decomposition research over the past several decades. For example, while there were only a few studies presented each year at the American Academy of Forensic Sciences annual meetings on decomposition a few decades ago, a review of the 2002–2018 *Proceedings of the American Academy of Forensic Sciences* reveals a growing interest in decomposition related studies (Figure 1). Between 2002 and 2005, for example, there were approximately 8–9 presentations per year focusing on decomposition, but from 2014 to 2018 the average skyrocketed to 34 presentations per year. Much of the decomposition-related work in forensic anthropology has focused on gross morphological changes of the body, regional variation, intrinsic and extrinsic influences, grave soil ecology, vegetation, the effect of scavengers to aid in PMI estimation, detection of clandestine remains, and scene or trauma interpretation. In the other forensic sciences, decomposition-related work has put emphasis on chemical changes (e.g. volatile organic compounds, soil chemistry) and insect and microbiological biodiversity and succession associated with the decomposition of carrion, especially as it relates to estimating the PMI and other forensic and non-forensic uses. This broadening of scientific questions in the forensic sciences led to an increase in the number of human decomposition facilities and a growth in interdisciplinary research focused on decomposition ecology. As a result, many recent advancements in the forensic sciences over the past several decades have been associated with decomposition research.

The purpose of this article is to review some of the scientific advances that have occurred in decomposition research and how they can be applied in forensic anthropology. While it is not possible to cover all the literature or topics on decomposition research, my goal is to provide the reader with a basic understanding of our current knowledge of human decomposition, some of the relevant historical developments, and how this knowledge is applied to forensic anthropological cases for the detection (i.e. search) of clandestine remains, documentation of the scene, and the estimation of the PMI. Because of the wealth of articles on the early postmortem interval (<48 h) when primarily biochemical processes are occurring, this article will mostly address changes associated with gross decomposition (i.e. post-autolysis).

The article is divided into several sections. In the first section, I discuss some of the fundamental shifts in the way we approach decomposition research (i.e. basic concepts, experimental practices, technology, and the use of theory) that have led to greater understanding of human decomposition and its application in the forensic sciences. Over the past several decades, there has been a greater emphasis on examining decomposition within an evolutionary and ecological context (carrion ecology), on interdisciplinary research, and to quantifying the process of decomposition and the factors that influence its rate. Advancements in decomposition research have also been greatly enhanced by the recent explosion in the number of human decomposition facilities and the development of new molecular sequencing technologies. This section will conclude with examination of research associated with increasing our knowledge of the major extrinsic factors that affect the pattern of decomposition and its rate of progression. Much of this research has focused on terrestrial decomposition. Less work has been done on aquatic decomposition, but significant advances into our understanding of decomposition in water have been made using case studies of human remains and actualistic studies based on animal analogs. In the second section of the article, I discuss how these advances have been applied to detection of clandestine remains and the estimation of the PMI, with a focus on methods relevant to forensic anthropologists. I conclude the article with a discussion of future needs and potential research areas.

## Fundamental shifts in decomposition research

### Decomposition facilities

The establishment of decomposition research facilities has brought about a new era in decomposition studies. The first facility, the Anthropological Research Facility (ARF), was established at the University of Tennessee in 1980 by Dr William Bass. Shirley et al. [2] and Vidoli et al. [3] provide good overview of the ARF for readers interested in its history. Beginning in the 2000s, several other facilities opened. Today there are seven facilities in the United States, one in Europe, and one in Australia (Table 1) and more are in the planning stages. These decomposition facilities provide interdisciplinary opportunities to conduct semi-controlled actualistic research to test specific hypotheses using large samples of human remains with known PMI and for comparisons of patterns and rates of decomposition between climatic and ecological zones. Prior to the increase in human decomposition facilities, most studies were retrospective case studies or actualistic studies conducted using surrogates, especially pigs. Many previous studies were also cross-sectional. Research at decomposition facilities allows for longitudinal studies which are more accommodating for theory building [4]. Longitudinal actualistic studies also allow researchers to gain a better understanding of the specific factors that control the patterns and rate of decomposition. Longitudinal studies also allow researchers to retrospectively examine factors such as disease, trauma, antibiotics, body size and others that may influence patterns and rates of decomposition in medicolegal investigations. Probably most significant, decomposition facilities have allowed for an increase in theses and dissertations on the topic of decomposition in numerous scientific fields, which have greatly expanded our knowledge of the decomposition process and factors that affect the pattern and rate of decay and the dispersion of nutrients from the carcass into the ecosystem. Also of great importance is that these decomposition facilities provide a resource for medicolegal death investigators, law enforcement, and students to train in burial excavation techniques, documenting scattered surface remains, and observing the decomposition process. These training opportunities encourage and assure better and more standardized evidence collection during outdoor scene recoveries.

**Table 1.** Human decomposition facilities.

Facility	Year established	Country	School	Department	Environment^a^
Anthropology Research Facility (ARF)	1981	United States	University of Tennessee	Anthropology	Temperate, without dry season and hot summers
Forensic Osteology Research Station (FOREST)	2007	United State	Western Carolina University	Anthropology	Temperate, without dry season and hot summers
Forensic Anthropology Research Facility (FARF)	2008	United States	Texas State University	Anthropology	Temperate, without dry season and hot summers
Applied Anatomical Research Center of Southwest Texas	2008	United States	Sam Houston State University	Criminal Justice	Temperate, without dry season and hot summers
Complex for Forensic Anthropology Research (CFAR)	2010	United States	Southern Illinois University	Anthropology	Temperate, without dry season and warm summers
Forensic Investigation Research Station (FIRS)	2012	United States	Colorado Mesa	Criminal Justice	Arid, steppe and cold
Australian Facility for Taphonomic Experimental Anthropologist Research (AFTER)	2016	Australia	University of Technology Sydney	Centre for Forensic Sciences	Temperate, without dry season and hot summers
Taphonomy Cemetery	2016	Holland	Amsterdam’s Academic Medical Center	Medicine	Temperate without dry season and warm summers
Florida Forensic Institute for Research, Security, and Tactical Training	2017	United States	University of Southern Florida	Anthropology	Temperate, without dry season and hot summers
Forensic Research Outdoor Station (FROST)	2018	United States	Northern Michigan University	Anthropology	Cold, dry winter, very cold winter

a Based on Köppen climate classification. However, there is considerable climatic variation within classifications.

#### Donated human remains

The need to conduct decomposition studies on human remains rather than animal surrogates to get forensically reliable data was realized by Dr William Bass, and more recent studies have confirmed that decomposition patterns and rates, microbial community distributions, and insect distributions differ between human and non-human animals [5–9]. To get forensically reliable data, there is a need to use human remains because scavenger diversity is closely tied to carcass size and possibly the microbiome present when the animal or person was alive [7–10]. Luckily in the past few decades, the number of human donations available for scientific research has grown considerably [11]. While most whole body donations in the United States are used for medical research and training, the number of individuals donating to human decomposition facilities has greatly increased. For example, at ARF whole body donations specifically for decomposition research have increased from a few individuals per year in the 1980s to over 100 bodies per year in the 2000s [3]. Currently the ARF has over 4 000 pre-registered donors, and interestingly more bodies are now declined than accepted [3]. Likewise, at Texas State University, body donations have increased from 3 per year in 2008 to over 70 per year in 2017 and will likely rise in the coming years as the number of pre-registered donations rises [12]. Currently, acquiring the funding necessary to conduct decomposition research is a larger obstacle than obtaining human bodies.

Bodies are donated to decomposition facilities through pre-registration by the donors themselves or next-of-kin donation by the family. These types of donations result in considerably greater biographical information about the life history and medical condition of the donors than receiving unclaimed bodies [3, 12]. When standardized decomposition data are collected on the donated remains these biographical data allow for retrospective studies based on large sample sizes that can be used to test hypotheses and situations associated with specific cases and to develop and validate forensic anthropological methods. In addition, during intake (procedures conducted when the body arrives at the decomposition facility) additional information such as blood cards, anthropometrics, hair and fingernail samples, and other baseline data are collected that can be used in future research.

### Interdisciplinary research

Another important shift that has benefited decomposition research in the forensic sciences is a greater emphasis on inter- and trans-disciplinary research. In many criminal investigations, locating clandestine remains and the estimation of the PMI are important objectives. As a result, practitioners of numerous disciplines (e.g. anthropology, botany, entomology, genetics, geoscience, medicine, microbiology) have focused their research on understanding the complexity of decomposition to develop more accurate and precise methods for estimating the PMI and detecting concealed remains. Additionally, research on decomposition is also important to public health, disaster management, cemetery planning, livestock carcass disposal, soil ecology, and more [13], and the information gained from studies in other fields is often directly relatable to the goals of forensic scientists. As Mondor et al. [14] point out, studying carrion decomposition not only allows us to understand how ecosystems function but can also be applied to solve medicolegal cases and to manage natural environments. Since decomposition is a complex issue there is a growing need for inter- or trans-disciplinary studies focusing on the evolution and ecology of decomposing human remains [15]. To fully understand the decomposition process and then apply this knowledge to forensic questions requires the use of theory and methodology from numerous disciplines [15].

### Carrion ecology

One of the major shifts that has benefited forensically focused research is to examine human decomposition using the theoretical foundation of carrion ecology. Since decomposition occurs in an ecosystem, to fully understand the decomposition process researchers interested in forensic applications will gain significant insight by examining the process within an ecological and evolutionary perspective and using the foundation of succession, coexistence, optimal foraging, and other theories to explain the spatial and temporal occurrence of necrophagous species [16–18]. Carrion ecology studies allow researchers to examine the “spatial and temporal effects of carrion on soil nutrients, microbes, plants, arthropods, and vertebrates” [18]. While decomposition ecology has long been a focus in biology, only in the past few decades have we examined human decomposition within an ecological and evolutionary context [16, 17, 19]. A grounding of human decomposition in basic empirical research using ecological theories not only strengthens our understanding of human decomposition but also improves accuracy and precision of the methods applied to forensic investigations [16]. Furthermore, the use of ecological and other theory in decomposition research directly addresses some of the criticisms and recommendations made by the National Research Council [20] to strengthen the forensic sciences.

Carrion (carcasses of once living animals including humans) provides a large variety of facultative scavengers with a nutrient-rich but short-term resource that has been conceptualized as an ephemeral resource patch [21] or a cadaver decomposition island [19]. Decomposition of carrion is a continuous process primarily carried out through chemical degradation and reduction of the carcass by several different organisms that consume the carrion and transform the organic materials. Since carrion is an ephemeral resource, numerous species have evolved strategies such as altered life history traits and behaviours to exploit the resource before it is consumed by other organisms [18, 22]. Since much of the mass of the carcass is removed by necrophagous species, gaining knowledge about how necrophagous species are attracted to carrion, their pattern of succession, and how the environment affects their growth, development, and biodiversity is key to understanding decomposition. In general, while the goals of forensic-focused decomposition studies are usually centred on using the decomposition process to discover clandestine remains, estimate the PMI, interpret trauma, or other applied applications, knowledge of carrion ecology will greatly advance our ability to accurately and precisely meet these goals.

### Technological advancements

Numerous recent scientific advancements from microbiology and metagenomics to computational and remote sensing technologies have significantly contributed to investigations of carrion ecology and its application in forensic sciences. As Benbow et al. [18] have noted, these advancements have led to “a better resolution of relationships among organisms assembling as a community around or on an ephemeral resource patch.” With the advancement of metagenomics, microbial species can be identified to the genus level and their function during decomposition can be better understood. It has been hypothesized that microbial community functional profiles change as different carbon sources become available. Other technologies such as geophysical resistivity (differences in electrical current in soils) and hyperspectral imaging have also increased our ability to detect clandestine graves.

### Quantifying gross decomposition

In the past several decades, forensic anthropology has also undergone a few major shifts in the way decomposition is viewed. Much of the early research focused on describing discrete categories of decomposition based on stages of decomposition and rates of decay in calendar days [23–28]. For example, Reed [23] developed a four-stage process of decomposition (fresh, bloat, decay, and dry) that was used by Rodriguez and Bass [25] in the first major study of human remains at ARF. Later, Payne [24] outlined a six-stage process based on pigs, further subdividing Reed’s [24] “decay” stage into “active” and “advanced” and adding a “remains” category as the final stage. Later, Galloway and colleagues [27, 28] examined the pattern of decomposition using a retrospective study of forensic cases from the Sonoran Desert and developed a five-stage classification that is still commonly used in forensic anthropology. They categorized decomposition as fresh, early decomposition, advanced decomposition, skeletonization, and extreme decomposition (i.e. destruction of the skeletal remains). Later research has demonstrated that there are unclear demarcations between stages of decomposition [29, 30] and considerable variation in progression due to regional, seasonal, and micro-environmental conditions [31, 32].

Since 2005, there have been several attempts to quantify the gross morphological changes in the body and to examine decomposition as a continuous process [29, 33–35]. One method is Megyesi et al.s’ [33] total body score (TBS) system based on the stages of decomposition defined by Galloway et al. [28]. These authors realized that there were progressive characteristics during each stage of decomposition and that differential rates of decomposition occur among the head/neck, torso, and extremities. Likewise, Fitzgerald and Oxenham [34] developed the degree of decomposition index (DDI) that provides a value between 0 and 5 based on the stage of decomposition for each body element present. More recently, Gleiber et al. [35] have been working to develop the accumulated decomposition score (ADS) that uses component scoring of traits based on gross observations of bodies in Texas. The ADS allows investigators to sum the traits observed rather than quantify the stage of decomposition.

The concept of using accumulated degree-days (ADD) or the sum of the average temperatures since deposition rather than calendar days was first introduced into forensic anthropology by Vass et al. [29]. However, this shift did not really take hold until the publication by Megyesi et al. [33]. The concept of ADD had already been used in many other sciences such as entomology, microbiology, and agriculture and provides a proxy measure for the energy available for decomposition processes that include chemical reactions and bacterial and insect growth and development. The advantage of ADD is that it incorporates chronological time and temperature and can hypothetically be used across different climatic regions and seasons.

Prior to 1992, most anthropologists described the rate of gross decomposition of the body in calendar days since death or placement. These early studies pointed out that there was considerable variation in the rate of decomposition depending on regional climatic differences due primarily to ambient temperature, insect colonization, deposition (surface, buried, aquatic), and burial depth [25, 26, 36]. For example, Rodriguez and Bass [25] observed that four bodies deposited on the ground surface were in a fresh stage from 4 to 36 d and in the bloat stage from 3 to 19 d depending on the season of placement. Likewise, Rodriguez and Bass [26] observed that bodies buried at a depth of approximately 30.48 m decomposed more rapidly than bodies buried at 60.96 or 121.92 m below the ground surface due to decreased insect access and cooler temperatures. In their study of six individuals at ARF, the body buried at a depth of 121.92 m retained considerably greater soft tissue after 1 year than a body buried at 30.48 m for 3 months.

While numerous validation studies have demonstrated problems with the methods developed by Vass et al. [29, 30] and Megyesi et al. [33], these works were significant because they caused a shift in the way anthropologists think about decomposition. Now, it is viewed as a process influenced by temperature and other environmental factors rather than stages that could be described in calendar days. A shift to using ADD rather than calendar days has allowed for a more realistic understanding of decomposition and has greatly improved the accuracy and precision of methods for estimating the rate of decomposition. These methods also included mathematical formulae to estimate the PMI and to provide error estimations (discussed in more detail below).

### Improved understanding of extrinsic factors

**Figure 2.**
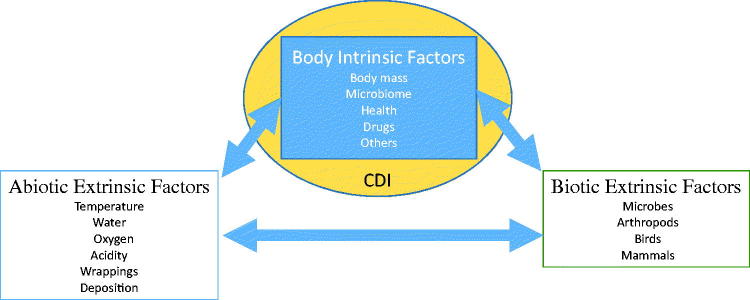
Intrinsic and extrinsic factors affecting the rate of decomposition. CDI: cadaver decomposition island.

At death, the human body begins to decompose and successively undergoes gross physical changes such as skin slippage, marbling, bloat, purge, and skeletonization, but the rate at which decomposition occurs is dependent on a number of intrinsic and extrinsic factors (Figure 2). Below I will discuss some of the more important extrinsic factors affecting decomposition.

#### Abiotic extrinsic factors

While many abiotic extrinsic factors can influence the rate of decomposition (e.g. soil type, clothing or coverings, concrete encasement, solar radiation, etc.), this is primarily because they influence the ambient temperature, acidity, availability of water, and the partial pressure of oxygen [28, 30, 33, 37–40]. These four extrinsic factors constraint the chemistry of decomposition (e.g., enzymatic breakdown of molecules) and the lifecycle of microbes and arthropods that influence the rate of decomposition. These environmental variables also greatly influence the preservation of tissues through desiccation and adiopocere formation. In some ways, the effects of these different environmental factors are difficult to separate and can influence each other. For example, water can affect the pH by acting as a buffer, stabilize temperature because of its high specific heat, and reduce the availability of oxygen [38].

*Temperature:* The ambient temperature in which human remains decompose is one of the most important abiotic extrinsic factors influencing the rate of decomposition. Temperature has a major influence on chemical reactions, the proliferation and metabolism of microbes, and the growth and development of necrophagous arthropods. In general, cadaver mass decreases more rapidly as the temperature increases. However, while the rate of chemical reactions generally increases two or more times with each 10 °C rise in temperature, the development of microbes, and the colonization and development of arthropods occur most rapidly within optimal temperature ranges [13, 38, 41–43]. For example, temperatures above or below the optimal conditions can reduce arthropod colonization and development.

The temperature is often not constant during various periods of decomposition and can be affected by multiple factors including the location (outside or inside, terrestrial or aquatic, climatic region, sunny or shady area, air movement, altitude), type of deposition (surface, buried, water), season of death, and microbial and arthropod biomass to name a few. For example, Rodriguez and Bass [26] observed a 3 °C–10 °C increase around the body compared with the surrounding soil even at 121.92 m below the ground surface. This increase in temperature around the human remains was greater than previous studies using animal carcasses [23, 24], and implies that the decomposition process generates heat that can cause the ambient temperature adjacent to the body to be higher than the surrounding air or soil temperatures.

*Water:* Water is also necessary for decomposition and can come from a variety of sources including humidity, precipitation, and waterbodies. As stated by Gill-King [38] “water plays both a diluting role, affecting chemical concentrations inside and outside cells, and acts, in general, as a solvent for polar molecules of biological and non-biological origin.” Water can increase or decrease the rate of decomposition depending on quantity, pH, and other factors [38, 44–46]. During decomposition, water from the soft tissues will either collect around the body or be removed due to humidity and soil moisture content [30]. Vass [30] argues that when moisture levels drop below 85% the rate of decomposition increases but when levels are greater than 85% decomposition rates decrease.

The primary influence of water on decomposition is most likely due to its effects on microbial activity. Optimal water content can increase microbial growth and proliferation, but above or below optimal moisture can retard microbial activity. Carter et al. [47] found that soil moisture was one of the primary environmental factors affecting the rate of decomposition in buried remains and had an influence on the relationship between temperature and decomposition. They found that decomposition was slower in dry soils because of a reduction in microbes and enzymatic reactions, but water saturated soils also decrease the aerobic metabolism of microbes and, therefore, decrease decomposition rates even when temperature was held constant. Carter et al. [47] argue that gas diffusivity in saturated soils affects aerobic metabolism while dry soils influence the availability of nutrients.

Under certain environmental conditions, decomposition nearly ceases due to the presence or absence of water. For example, moisture plays a role in desiccation/mummification of the remains and the formation of adiopocere, which is a byproduct of lipid degradation. Dry, well-drained soils, and arid environments [hot or cold] are favourable to desiccation while moist and microbial-rich environments are conducive to adiopocere formation [38, 48, 49]. However, longitudinal research in central and eastern Texas as well as Tennessee demonstrate that bodies left on the ground surface often form a desiccated shell of skin around the otherwise skeletal remains even though all these environments are considered subtropical humid [50]. To investigate the causes of this phenomenon, Lennartz [51] conducted a pilot study examining desiccation and mummification of skin in central Texas. She specifically examined the effects of temperature, humidity, precipitation, and solar radiation on moisture changes in the skin. Her results showed that the skin loses moisture rapidly during the first 1 000 ADD but becomes mummified at approximately 10% moisture content when changes become asymptotic. She also discovered that temperature was the most important factor in the prediction of moisture loss. In her study, there was approximately a 9% loss in moisture with each 10 °C increase in temperature. Surprisingly, Lennartz [51] found no significant correlation between desiccation rates and humidity, precipitation, or solar radiation.

Decomposition of submerged bodies is generally slower than in terrestrial environments due to cooler temperature and the reduction of insects [46]. However, the rate of decomposition is highly dependent on numerous factors: if the body is in fresh or salt water, if the water is stagnated or flowing, the types of flora and fauna present, and the water temperature and pH. Furthermore, the general stages in the process of decomposition differ slightly for bodies completely submerged compared with bodies in a terrestrial environment. For example, decomposition stages in aquatic environments are frequently categorized as fresh submerged, early floating, early floating decay, advanced floating decay, and sunken [46, 52–54].

*pH and oxygen:* The acidity/alkalinity of soils and the partial pressure of oxygen can affect the rate of decomposition. The pH has its greatest influence on chemical reactions during decomposition. However, the pH is also temporarily influenced by the decomposition process and water content [30, 38]. Surface decomposition is often alkaline due to aerobic conditions while burials are commonly acidic due to the liberation of organic acids by bacteria [30, 38]. Lower pH (acidity) can enhance the growth of fungi and plant activity. Research has demonstrated that decomposition is generally more rapid in the presence of oxygen. Therefore, bodies that are buried, submerged, or at high altitudes tend to decompose slower than the decomposition of bodies on the surface because oxidative processes are retarded [38]. However, the depletion of oxygen initiates decomposition and supports the activity of bacterial decomposers within and around the body.

#### Biotic extrinsic factors

*Microbes:* Bacteria are the first colonizers of decomposing carrion because these microorganisms are present at death. During putrefaction, bacteria and other microorganisms proliferate and play a vital role in the recycling of carrion through enzymatic degradation of tissues [10, 22, 55, 56]. The role of microbes in decomposition has been reviewed extensively elsewhere [13, 55–64], but the research has demonstrated that understanding microbial population taxonomic and functional succession can provide significant insight into the decomposition process. Numerous studies have shown that the microbial decomposer community diversity and function (metabolism) progressively change during decomposition in a predictable fashion [10, 55, 56, 59]. In general, aerobic microorganisms use the oxygen available in tissues, but as oxygen becomes depleted the environment favours anaerobic microorganisms. As the body dries the microorganism community decreases in abundance, but soil bacteria that produce collagenase and keratinase remain active [55]. Cobaugh et al. [55], for example, demonstrated that in buried remains the microbial community changed during active decay with an increase in the relative abundance of aerobic bacteria such as Proteobacteria and Firmicutes but a reduction in Acidobacteria. After active decay, the microbial community is dominated by anaerobic taxa.

Bacteria are also responsible for many aspects of decomposition. Bacteria produce gas byproducts such as methane, cadaverine, putrescine, hydrogen sulphide, and ammonia within the body that cause bloating and affect the pH of the body and local scavengers and plants. Furthermore, bacteria appear to manipulate the behaviour of insects to attract species that benefit their survival while repelling those that are detrimental to them [22]. The volatile organic compounds (VOCs) produced as bacteria degrade carrion are responsible for attracting blow flies to colonize. Additionally, the presence of bacteria species may be necessary for proper development of many fly species [65, 66]. Therefore, knowledge of bacteria succession and function during decomposition through metagenomics research is important to understand the downstream effects on decomposition rates and patterns.

*Arthropods:* Much of the research on decomposition outside of anthropology has focused on necrophagous insects, especially flies and beetles, which are a major contributor to biomass reduction. Factors affecting colonization and the lifecycle of these species have been the primary emphasis of research and are discussed in detail in numerous publications [16, 66–81]. Tomberlin et al. [16] describe the ecologically relevant temporal and physical aspects of insect activity. They argue that entomological activity can be divided into pre-colonization and post-colonization intervals.

The pre-colonization interval includes the exposure, detection, and acceptance phases beginning when carrion is available and then detected by arthropods and lasts until it is accepted or rejected as a resource. The exposure phase is difficult to estimate and can be affected by any factor that limits it. Once carrion is detected, environmental conditions such as wind speed, precipitation, temperature, humidity, as well as mating status and ovarian development affect the response of arthropods to carrion [16]. During the acceptance phase, “arthropods use close-range cues including colour, shape, size, movement, sound, and taste to evaluate the resource” to determine the suitability of the carrion [16].

The post-colonization period involves the consumption and dispersal phases and lasts from the initial colonization until departure from the carrion after complete decomposition or the removal of the carrion source. The post-colonization period is a good predictor of the minimum PMI or the period of insect activity [16]. The consumption phase involves successive waves of insects or their offspring feeding on the carrion. Development of the offspring is primarily used to estimate the length of the consumption phase. Finally, once developed, the insects will depart from the remains to complete their lifecycle. However, abiotic factors and disturbance of the carrion can cause premature departure.

In addition to understanding the temporal succession of arthropods and knowing some of the major species (e.g. blow, flesh, green bottle, and soldier flies and carrion and dermestid beetles), there are a few other important aspects of entomology that forensic anthropologists should keep in mind. First, colonization may not coincide with the PMI since colonization can occur long after death or sometimes before death. Second, many of the insects present on human remains are feeding on other insects and not scavenging on the cadaver. Third, numerous biotic and abiotic factors can influence insect activity and development. Finally, research has demonstrated that the composition, not just the abundance of insect scavengers, is key to the rate of decomposition [16, 78].

*Large scavengers:* Besides microbes and insects, the effects of mammalian and avian scavengers on decomposition have been studied [82–98]. Most studies have focused on larger mammals and birds, but some have also examined the effects of reptiles and small mammals. While larger scavengers are a primary extrinsic factor in the decomposition process, most scavenging by larger mammals and birds are opportunistic. In the United States, vultures are the only obligated carrion feeders.

Most of these studies on the effects of animals have examined the role of scavengers in the removal of soft tissues, disarticulation, and scattering, which affect the search and recovery of human remains as well as the estimation of the PMI. Vultures, for example, will typically begin to feed on carrion during early putrefaction and can consume much of the soft tissue within hours [87, 90, 91, 93, 98]. However, vultures typically do not remove or scatter remains more than a few meters from the original placement location [90, 93, 94, 98].

An important aspect of the influence of scavengers that has been largely ignored is the examination of the behaviours of the animals themselves. Haglund [99] and Pharr [94] have observed that the presence or absence of animal scavenging is associated with human population density and behavioural ecology of the scavengers. Haglund [99] argues that human population density can play a major role in whether large scavengers will exploit carrion because the remains are likely found earlier in urban than rural areas and there are fewer species and smaller group sizes of animals in populated areas. In her studies of the feeding behaviour of vultures in Texas, Pharr [94] observed that turkey and black vulture scavenging locations are on average within 450 m and 361 m, respectively, from a permanent water source. These results suggest that permanent waterbodies near the carrion may be necessary for larger scavengers.

## Applications of decomposition research

### Detection of clandestine remains

While human remains are often found by accident or through the use of informants, especially those not buried, organized search efforts are often necessary to locate clandestine graves and surface remains. In these cases, the end results of decomposition are often used to help locate the remains. In reality, the search for concealed human remains often involves the search for disruptions in the natural environment caused by the decomposing corpse. Decomposing remains will have an effect on the vegetation and soil characteristics and will produce odours that can be used to help in their detection.

#### Vegetation and fungi

Plant composition provides information about underlying ecological conditions, and several studies have suggested that vegetation composition can be used to detect clandestine graves [26, 100–102]. Visible differences can often be detected between the dominant weed flora on graves and surrounding cadaver decomposition islands (CDIs) compared with the undisturbed surrounding soils. Likewise, Carter and Tibbett [103] found that the presence of post-putrefaction fungi on graves in wooded areas may also be used in grave detection.

The process of burial itself disturbs the soil and overlying vegetation at the site. For surface remains the release of cadaveric fluids that form the CDI, which are high in ammonia, will initially kill surrounding vegetation. Over time, pioneer plants will begin to colonize the grave soils and the edges of the CDI as nutrients, especially nitrogen and phosphate, are converted by soil bacteria into a usable form [19]. However, eventually the plant composition will once again become similar to that in the surrounding areas [100]. Interestingly, while decomposition is known to change the characteristics of grave soil (temperature, moisture, and nutrients), the aeration of the disturbed soils may have a greater impact on plant colonization than does nutrient enrichment [100, 104, 105].

#### Remote sensing (imagery)

Several studies have used remote sensing to help locate remains by examining environmental disturbances caused by the decomposing carcass. Remote sensing can provide a rapid and cost-effective method for determining high probability areas during the initial search [106, 107]. Methods for locating remains using remote sensing are in part determined by the state of decomposition, geographical location, and deposition type. Kalacska et al. [108, 109] examined the use of remote sensing using airborne hyperspectral imaging and discovered that mass graves in a tropical moist environment have a distinct spectral signature based on the spectral response to decomposition products. Similarly, Isaacks [110] determined that remote sensing using unmanned aerial vehicles (UAV) equipped with near-infrared (NIR) sensors could be used to effectively and expediently locate surface depositions for up to 2 years based on differences in the reflectance of the surrounding area and the CDI. Cadaveric fluids purged out of the decomposing body seep into the soil causing it to become organically rich, which produces a different spectral signature in NIR than the surrounding soil and vegetation. Isaacks [110] and Kalacska et al. [108, 109] also discovered that the spectral signature changes as plants recolonize the soils but the signature remains distinct from the undisturbed areas and disturbed soils without carcasses. Current work by Wescott et al. [106, 107] is examining the best platform and spectral bands (e.g. NIR, long-wave infrared) to detect anomalies and the potential development of a graphical user interface to aid search teams in locating buried and clandestine surface remains.

#### Human remains detection dogs and VOCs

During soft tissue decomposition, a variety of compounds including volatile organic compounds (VOCs) are produced and are responsible for the odour of decomposition [111, 112]. Research into the VOCs produced during decomposition can provide information to help detect concealed remains as well as estimate the PMI. Human remains detection (HRD) dogs, for example, detect VOCs. In a series of publications, Vass et al. [111, 113, 114] examined the chemicals associated with the odour of decomposition and the development of the “Decomposition Odour Analysis (DOA) Database.” These and other studies [115–118] demonstrated that the chemicals associated with decomposition change over time. Vass [111] concludes: “Currently it is not yet possible to accurately predict which compounds will be present at any given decompositional event since the mechanisms of compound formation and the taphonomic influences are not yet fully understood.” However, Carabollo [119] found that examining the type and abundance of compounds in the total odour profile can be used to distinguish each stage of decomposition. The early decomposition/bloat stage and the active decay stage showed the least amount of variation in the compounds present and their per cent of the total composition.

Ideally, determining human-specific compounds present during decomposition will aid in the development of training aids for HRD dogs and the development of detection instrumentation. However, significant research is still needed because it is difficult to determine how the odour profile will change under different situations and postmortem intervals. Caraballo [112], for example, documented that the decomposition environment influences the odour released by enhancing or hindering the amount of odour liberated, and that skeletonized remains do not have a unique VOC profile. Dekeirsschieter et al. [116] found that the VOCs of bodies decomposing in urban settings differed significantly from those in open air outdoor sites, and pollutants in the air caused background noise that is difficult to separate.

### Postmortem interval

Forbes [49] has pointed out that PMI estimation is one of the more elusive aspects of any medicolegal death investigation. This is in part because there is considerable unpredictability in the rate at which decomposition progresses in human remains, especially with increasing PMI. Unfortunately, it is also in part because of the current state of research in decomposition. As observed by Passalacqua and Megyesi [119], over 60% of the studies in the *Journal of Forensic Sciences* [1972–2014] and the American Academy of Forensic Sciences (AAFS) *Proceedings* [2002–2014] examining the PMI were either descriptive or described unique settings and over 75% used animal surrogates. In addition, the methods used to estimate the PMI frequently vary depending on the progression or stage of decomposition.

Regardless of the method used to estimate the PMI there are important criteria necessary for the method to gain wide-spread acceptance among practitioners. Henssge and Madea [120] argued that any method for estimating the PMI will “only gain practical relevance if the following criteria are fulfilled: quantitative measurement, mathematical description, taking into account influencing factors quantitatively, declaration of precision and proof of precision on independent materials.” Below I will discuss some of the methods used to estimate the PMI based on gross morphological changes commonly used by forensic anthropologists. Information on microbial biodiversity and succession to estimate the PMI as well insect colonization, development and succession to estimate the time-since-colonization has been extensively reviewed elsewhere [121–125].

In the past several decades, a few methods based on gross physical changes in the body have attempted to meet the vital criteria outlined by Henssge and Madea [120]. Probably the most commonly used method for estimating the PMI based on gross physical changes in the human body was developed by Megyesi et al. [33]. As discussed earlier, their method attempts to quantify the stages of decomposition through a point-based system or total body score (TBS) and correlate it with ADD. Using this method, investigators score the gross decompositional changes of three anatomical regions (i.e. head/neck, torso, extremities) and sum the scores to obtain a TBS. Scores depicting changes occur from fresh to dry bone and range from 1 to 13 for the head/neck, 1 to 12 for the trunk, and 1 to 10 for the extremities. Therefore, the TBS can range from 3 to 35. The total decomposition score is then inserted into a regression equation by investigators to calculate the ADD necessary for the body to reach the observed stage of decomposition for the remains under investigation. Investigators use ambient temperature data from a nearby national weather station to calculate the most likely date of death based on the “local” ADD. The advantage of the TBS/ADD method is that it meets most of the criteria outlined by Henssge and Madea [120]. The method uses a quantitative measure, mathematical description, considers the influence of temperature on the quantitative measure, and provides a quantitative measure of error. Furthermore, several studies have demonstrated high interobserver reliability in the quantifying decomposition using the TBS [50, 126].

Later, Vass [30] proposed two formulae for estimating the time since death for surface (aerobic) or buried (anaerobic) remains. Unlike the method proposed by Megyesi et al. [33] that only considers temperature variation, Vass [30] argued that temperature, moisture, pH, and partial pressure of oxygen should be accounted for in a PMI estimation equation. The method devised by Vass [30] for surface remains uses a constant ADD of 1 285 multiplied by the percentage of soft tissue remaining as the numerator and multiplies the average temperature, average humidity, and a constant of 0.010 3 for moisture in the denominator. The results of this equation provide an estimation of the PMI in calendar days. For buried remains, Vass [30] used the 1 285 ADD constant multiplied by a 4.6 constant for the lack of oxygen and the percentage of adipocere as the numerator. The denominator includes the constant 0.010 3 to represent the moisture effect on decomposition multiplied by the soil temperature and soil moisture. Like the TBS/ADD method, this “universal” method meets most of the criteria proposed by Henssge and Madea [120] needed for a relevant PMI estimation method, although it does not provide an error rate. Furthermore, inter-observer error in estimating the percentage of decomposition has not been evaluated.

Much of the work since the development of the methods by Megyesi et al. [33] and Vass [30] has been associated with validation and improvement of these methods. Unfortunately, the one criterion stated by Henssge and Madea [120] that both methods have failed is for “proof of precision on independent materials.” Numerous studies have demonstrated these methods do not accurately or precisely estimate the PMI, especially as PMI advances or in extreme environments [50, 127–129]. Research has demonstrated a need for regional formulae that take into account climatic variables as well as different formulae depending on the scene context (indoor, outdoor, surface, buried, aquatic, clothed, unclothed), body position (hanging, burial depth), body condition (burned, cause of death, etc.), and individual characteristics of the cadaver (age, sex, body weight, and microbiome). As a result, there have been numerous calls for region-specific equations [31,130–132] and season of death [133] as well as equations for aquatic deposition [45, 134, 135], hanging [136], and charred remains [137]. In aquatic deposition remains, for example, the total aquatic decomposition (TAD) can be used in combination with ADD (based on thermal energy available in the water) to provide a quantitative method for estimating the postmortem submersion interval (PMSI). Like the TBS, the TAD examines changes in the head, body, and limbs [45].

Several researchers have also examined statistical aspects of calculating the PMI for terrestrial remains including Michaud and Moreau [138] and Moffatt et al. [139]. Michaud and Morea [138] used different minimum ADD thresholds rather than just the average above zero temperature used by Megyesi et al. [33] and Vass [30]. This method accounts for more variability in decomposition rates, but most importantly it provides probabilities associated with each stage of decomposition. Noting problems with the statistical methods used by Megyesi et al. [33], Moffatt et al. [139] developed a new formula based on inverse regression for estimating the ADD from TBS that provides smaller predictive intervals. Unfortunately, there have been few attempts to validate the revised methods presented by Michaud and Moreau [138] and Moffatt et al. [139].

Overall, gross morphological changes to the body have been the primary focus of anthropological work to estimate the PMI. Over the past several decades, significant advances have been made to quantify the decomposition process and to account for some of the variables, primarily temperature that influences the rate of decomposition. While there are still numerous problems with the accuracy and precision of the methods, work by Megyesi et al. [33], Vass [30] and others have advanced the way we approach the estimation of the PMI.

## Future needs

The research on decomposition is still in its scientific infancy. In the future, there is a greater need for the collection of standardized data, more rigorous statistical analyses, examination of extended PMIs, greater emphasis on aquatic decomposition and carrion ecology, interdisciplinary or transdisciplinary research and the use of human cadavers to get forensically reliable data. Some of the problems associated with decomposition research are that there are limited datasets available for study and comparison, as well as a general lack of standardized nomenclature, multi-regional comparative studies, and true inter- and trans-disciplinary research. Probably, most important to decomposition research is the need for greater use of theory in decomposition research and the development of trans-disciplinary theory. Boyd and Boyd [140], for example, discuss the use of non-linear systems theory to improve estimates of PMI based on gross physical characteristics. Likewise, the use of more rigorous statistical methods such as mixed-effect models, transition analysis, and others are needed. Wescott [15] has also called for a greater need of trans-disciplinary research that incorporates methodology and theory from numerous disciplines including ecological and evolutionary theory in all decomposition studies. Furthermore, there is a need to gain a basic understanding of decomposition ecology instead of focusing on a wide variety of factors that could influence the decomposition rate. Likewise, while multiple studies show great promise for examining soil chemistry [9] and VOCs [112], these studies need to be examined within the larger ecological and evolutionary context. While the study of microbiology has increased in the past several decades, the examination of the effects of the microbiome of the deceased individual and how it influences decomposition will go a long way towards increasing our knowledge of decomposition. Finally, there is a need to examine the intrinsic factors of the body that affect decomposition.

## Conclusion

Decomposition research has provided forensic anthropology with a new conceptional framework that is grounded in the natural sciences. We now have a greater understanding of the complexity of decomposition and the variability caused by numerous biotic and abiotic variables that affect the rate and pattern of progression in human remains. While in some ways research over the past several years has demonstrated the unpredictability of decomposition, the research holds promise for developing better methods for the detection of human remains, interpretation of scenes, and the estimation of the postmortem interval. However, because of the uncertainty in decomposition, many forensic anthropologists are still leery about interpreting decomposition to estimate the PMI, but understanding this unpredictability and when and why we can or cannot make accurate or precise estimations of the PMI is also critical to medicolegal death investigations. I have no doubt that as we continue to explore the mechanisms of decomposition through an ecological and evolutionary perspective that we will also develop more accurate and precise methods that utilize quantitative measures and known error rates. While there have already been significant advancements in our knowledge of decomposition, I believe that even greater advancements are just around the corner.
